# Impact of green infrastructure in smart older adult care communities on the health of the older adult and the exploration of optimization paths

**DOI:** 10.3389/fpubh.2025.1601102

**Published:** 2025-06-09

**Authors:** Zhuang Wang, Lianping Gao, Peijuan Song

**Affiliations:** ^1^Journalism and Communications College, Jilin Normal University, Changchun, Jilin, China; ^2^College of Art and Design, Changchun Guanghua University, Changchun, Jilin, China

**Keywords:** green infrastructure, older adult health, influencing factors, path optimization, smart older adult care community

## Abstract

**Introduction:**

This study explores the multidimensional impacts of green infrastructure (GI) within smart older adult care communities on the physical health, psychological wellbeing, and social interactions of older adults. It further investigates optimization strategies for GI design to support healthy aging policies and enhance urban resilience.

**Methods:**

Using longitudinal panel data from the China Health and Retirement Longitudinal Study (CHARLS, 2012-2020), the study applies panel regression models to examine the association between GI coverage and various health outcomes among older adults. Heterogeneity analysis assesses demographic-specific effects, and structural equation modeling (SEM) evaluates the mediating pathways through which GI influences public health.

**Results:**

Regression results indicate that increased GI coverage significantly reduces the incidence of chronic and acute diseases (−0.34^**^), alleviates depression (−0.14), and anxiety (−0.12), and enhances subjective wellbeing (0.45) and frequency of social interactions (0.29^**^). Heterogeneity analysis reveals that health benefits are more pronounced among males and adults aged 70–79. SEM results show that GI indirectly mitigates the prevalence of COVID-19 by improving air quality (−0.15) and regulating ambient temperature (−0.10). Accessibility and equitable distribution of GI further amplify these positive effects.

**Discussion:**

The findings underscore the critical role of GI as a public health intervention in smart older adult care communities. By integrating environmental health mechanisms with smart technologies, the study offers actionable recommendations for optimizing GI design-such as improving spatial equity, connectivity, and microclimate resilience. These insights contribute to the development of age–friendly urban planning frameworks and enhanced pandemic preparedness strategies. The study advocates for policies prioritizing high-quality, accessible green spaces to promote health, advance social equity, and address climate adaptation challenges.

## 1 Introduction

As an innovative older adult care model that integrates advanced information technology and efficient community management, smart older adult care communities are becoming increasingly popular around the world and have become a research hotspot. With the intensification of the aging population problem (1, 2), health management has become an important issue in social development. Traditional older adult care services (3, 4) are limited by uneven resource distribution, insufficient service coverage, and low matching with the needs of the older adult. More scientific and efficient solutions are urgently needed. A smart older adult care community in Haidian District, Beijing, has increased its green space coverage through the construction of a “15-min green living circle” and simultaneously equipped it with an intelligent air monitoring system, which has reduced the incidence of chronic diseases among the older adult compared to traditional communities, verifying the strong correlation between green infrastructure and health promotion. In this context, green infrastructure (5, 6) has gradually been regarded as an important part of the construction of smart older adult care communities due to its multiple roles in improving community environment, promoting residents' health, and enhancing social interaction. The optimization of green infrastructure (7, 8) can relieve the physical and mental stress of the older adult by providing high-quality natural space, and has a significant effect on reducing the prevalence of chronic diseases, improving health levels, and enhancing subjective wellbeing. However, most existing studies focus on the single function or single dimension of green infrastructure. For example, the exploration of its health benefits is relatively extensive, but there is still a lack of systematic research on the multi-dimensional comprehensive effects of green infrastructure in smart older adult care communities. Especially in the optimization design of green infrastructure, there is a lack of in-depth analysis of its comprehensive effects in multiple dimensions such as health, social interaction, and psychological wellbeing, resulting in the failure of green infrastructure design in smart older adult care communities to fully consider the all-round impact of these key factors on the quality of life of the older adult. Therefore, the multidimensional role of green infrastructure (9, 10) and its application in smart older adult care communities are still a research gap that needs to be addressed.

In this paper, independent variables refer to key factors that can directly or indirectly affect the health level and social effects of the older adult, including green space coverage, green space distribution uniformity, green space connectivity, and green space accessibility. Independent variables play a decisive role in the causal relationship model, and their changes can cause corresponding changes in dependent variables (such as CHARLS disease index, Self-Rating Depression Scale (SDS), Self-Rating Anxiety Scale (SAS), Subjective Wellbeing (SWB) and interaction frequency), thereby reflecting the health promotion effect of green infrastructure in smart older adult care communities. Green infrastructure reduces the risk of disease in the older adult through direct effects and forms a complete causal chain through intermediary links such as improving air quality and regulating ambient temperature, thereby indirectly transmitting the impact. This paper uses a panel data regression model to quantitatively analyze the direct effect, and the results show that there is a significant negative relationship between green space coverage and various health indicators. At the same time, the structural equation model (SEM) is used to further explore the impact mechanism of green infrastructure on the prevalence of COVID-19 through two intermediary paths of air quality and ambient temperature. Through the systematic definition and empirical test of causal variables and their internal relationships, this study deepens the theoretical understanding of the mechanism of green infrastructure and provides a solid theoretical basis and methodological support for the planning of smart older adult care communities and the formulation of healthy aging policies.

The main contributions of this study are reflected in the three levels of theoretical construction, methodological innovation and practical guidance. Regarding theoretical construction, this paper constructs a comprehensive theoretical model of the impact of green infrastructure on the health of the older adult in smart older adult care communities, and systematically explains that green space coverage, distribution uniformity, connectivity and accessibility affect the physical health, psychological state and social interaction of the older adult, respectively, enriching the theoretical system in the field of healthy aging and urban green planning. In terms of methodological innovation, the study comprehensively uses panel data regression models and structural equation models, which quantitatively analyzes the direct relationship between green infrastructure and health-related indicators and introduces mediating variables such as air quality and ambient temperature to reveal the internal transmission mechanism of the impact of green infrastructure optimization design on the prevalence of COVID-19, and achieves accurate measurement of multi-level and full-path causal effects. In addition, through robustness tests of multiple time windows from 2012 to 2020 and heterogeneity tests for different genders and age groups, this paper further verifies the differences in the role of green infrastructure in different groups, providing a detailed basis for subsequent policy formulation. The research results provide scientific empirical support for the planning and construction of smart older adult care communities and the formulation of healthy aging policies. It clarifies the importance of increasing green space coverage and optimizing green space layout in reducing the risk of disease and improving subjective wellbeing among the older adult and puts forward specific suggestions for optimizing green space distribution, enhancing connectivity and improving accessibility, providing practical and feasible operational guidance for urban planners and public health decision makers, which has important theoretical value and practical significance.

## 2 Literature review and theoretical mechanism

### 2.1 Literature review

In recent years, China has carried out a number of innovative practices in coping with population aging and integrating green infrastructure. For example, a smart older adult care community in Haidian District, Beijing, has increased the green space coverage rate of the community by building a “15-min green living circle” and equipped with barrier-free trails and older adult-friendly recreational facilities. Studies have shown that the incidence of chronic diseases among the older adult in this community is significantly lower than that in traditional communities (11). Its technical solution of isolating PM2.5 through vegetation barriers has reduced the risk of respiratory diseases (12). Jing'an District, Shanghai, has piloted the “vertical greening + intelligent monitoring” model, setting up modular green walls on the facades of older adult apartments and combining IoT sensors to control the microclimate in real time, significantly alleviating the symptoms of cardiovascular diseases in the older adult (13). The smart older adult care community in Tianhe District, Guangzhou, has promoted the increase of the average daily outdoor activity time and social frequency of the older adult group by optimizing the Gini coefficient of green space distribution (14). These practices show that in the design of green infrastructure suitable for the older adult, China is using the three paths of spatial equity optimization, intelligent technology integration and ecological function enhancement to form a localized solution that takes into account both health promotion and epidemic prevention and control.

As an important innovative model to cope with the aging population, smart older adult care communities have received extensive attention in recent years. On the technical level, cutting-edge technologies such as big data, the Internet of Things, and artificial intelligence (15, 16) are widely used in the construction of smart older adult care communities to improve service efficiency and the accuracy of resource allocation. Community environment (17, 18) has become a new research direction. In this context, green infrastructure (19–21) has gradually been incorporated into the planning and design of smart older adult care communities due to its significant environmental benefits and health-promoting effects. Existing studies have shown that green infrastructure (22, 23) can not only optimize the living environment by improving air quality and reducing noise pollution, but also enhance the physical and Mental Health (MH) of community residents by providing natural space, promoting social interaction, and relieving psychological stress. However, most existing studies focus on the overall urban environment or ordinary communities. Research on green infrastructure specifically targeting the specific scenario of smart older adult care communities is still relatively scarce, and the discussion on its optimization path is limited to the theoretical framework and lacks systematic empirical analysis support.

Research on the health effects of green infrastructure mainly focuses on three dimensions: PH, MH, and social effects. Green infrastructure can reduce the incidence of chronic diseases by improving air quality (24, 25), reducing the urban heat island effect (26, 27), and increasing opportunities for exercise. Community environments with high greening rates (28, 29) have been shown to effectively reduce the risk of chronic diseases such as hypertension, heart disease, and diabetes among residents. The presence of green space (30, 31) has a significant effect on relieving stress, anxiety, and depression. Through exposure to natural landscapes, residents can benefit from psychological relaxation and emotional regulation, and their subjective wellbeing is significantly improved. Green infrastructure can also enhance the social effects of residents by promoting social interaction and enhancing a sense of community belonging. Open green space design (32, 33) helps the older adult participate in community activities and increase the breadth and depth of their social support network. However, these studies mostly focus on the single-dimensional functions of green infrastructure, and there is still a lack of discussion on its multi-dimensional interactive effects and health promotion mechanisms. Especially in smart older adult care communities, the older adult, as a special group, have more complex health needs.

Although the health effects of green infrastructure have been confirmed by a large number of studies, there are still significant gaps and challenges in the application research in smart older adult care communities. The planning and design of green infrastructure (34, 35) needs to take into account the physical, psychological, and social needs of the older adult, but existing research has paid little attention to the coordinated optimization of these multi-dimensional needs. The specific impacts of green space distribution uniformity, connectivity, and accessibility in different community scenarios are still lacking in systematic discussion. In the specific environment of smart older adult care communities (36), there is no clear theoretical framework or empirical support for how green infrastructure can indirectly affect the health of the older adult through mechanisms such as air quality improvement and thermal environment optimization. Existing research methods have certain limitations in data sources and analysis techniques. Most studies focus on a single time point or region, lacking long-term, cross-regional dynamic analysis. In particular, research on smart older adult care communities in China (37, 38) lacks systematic discussions based on high-quality data and advanced analytical methods. Research on the path of green infrastructure optimization still needs breakthroughs in theory and practice. How to scientifically measure the distribution uniformity, connectivity and accessibility of green space, and how to link these indicators with the health effects of the older adult, are still key issues that need to be solved. This study intends to provide a comprehensive and scientific theoretical basis and practical guidance for the optimization of green infrastructure in smart older adult care communities by constructing a theoretical model of green infrastructure optimization design and health effects, combined with large-scale empirical data analysis.

### 2.2 Theoretical mechanism

The high coverage rate of green infrastructure can significantly reduce the incidence of chronic and acute diseases in the older adult by improving the quality of the natural environment and enhancing physical activity opportunities. Green infrastructure improves air quality through vegetation cover (39), reduces the concentration of suspended particulate matter and harmful gases, and thus reduces the risk of respiratory and cardiovascular diseases. Green space (40, 41) can provide a safe and comfortable place for the older adult to exercise, encouraging them to engage in moderate outdoor activities such as walking, yoga, or tai chi, which can help improve cardiopulmonary function and bone health, and prevent chronic diseases such as obesity and diabetes. The coverage rate of green infrastructure not only promotes the PH of the older adult by directly optimizing the physical characteristics of the living environment, but also indirectly improves the quality of their lifestyle and improves their overall health from multiple dimensions.

**Hypothesis 1:** Based on environmental health theory, this study believes that smart older adult care communities with a high coverage rate of green infrastructure can effectively reduce the concentration of harmful substances in the environment by improving air quality, regulating microclimate, reducing noise pollution, etc., thereby reducing the risk of chronic diseases (cardiovascular diseases, respiratory diseases, etc.) and acute diseases among the older adult. In addition, high coverage of green space can encourage the older adult to participate in moderate outdoor sports and leisure activities, enhance physical fitness and immunity, and further improve overall health.

Green spaces, as public communication platforms, provide more opportunities for face-to-face interactions among older adults, helping to strengthen relationships and social connections among neighbors. Open and connected green infrastructure design (42, 43) can promote collective activities among community members, such as gardening clubs, fitness groups, and cultural activities, which not only increase the frequency of social interactions among older adults, but also enhance their sense of social belonging and the quality of their support networks. Green infrastructure can also provide more equal and accessible conditions for participation for the older adult by reducing community isolation and spatial barriers, thereby attracting more older adult people to participate in social activities. The optimization of green infrastructure is not only a reflection of the improvement of the community environment.

**Hypothesis 2:** From the perspective of social ecology and environmental psychology, a sound green infrastructure improves the physical living environment and builds a natural social platform. High-quality green space design (including walking paths, gardens, and public rest areas) in smart older adult care communities can promote natural communication and emotional interaction among the older adult and increase the frequency of interaction. Such environmental improvements can help enhance the older adult's sense of social participation and social support, and reduce loneliness and social isolation.

The optimal design of green infrastructure, including distribution uniformity, connectivity and accessibility, plays an important regulatory role in the MH of the older adult. Evenly distributed green spaces provide the older adult with a more convenient way to relax and regulate their emotions by shortening the physical distance of their daily contact with nature. Green connectivity (44, 45) can create continuous leisure and activity venues for the older adult by optimizing the spatial connections between green spaces, reduce the sense of environmental fragmentation, and enhance their psychological security and happiness. Accessibility, as one of the core factors in green infrastructure design, directly determines the actual convenience of the older adult in accessing the green environment. Shorter walking time and barrier-free design can effectively reduce the psychological burden of the older adult with limited mobility, making it easier for them to access the natural environment to alleviate depression and anxiety.

**Hypothesis 3:** In green infrastructure design, distribution uniformity, connectivity, and accessibility constitute the core indicators for optimizing layout. These design features ensure that green space resources are fairly distributed in space, so that every older adult person can easily access high-quality natural experiences. Connectivity and accessibility improve the frequency and quality of the older adult's daily use of green space by forming a continuous and barrier-free green space network, thereby effectively alleviating their depression and anxiety and improving their subjective wellbeing.

Optimizing green infrastructure design, especially in terms of distribution uniformity, connectivity and accessibility, is crucial to alleviating depression and anxiety among the older adult. Distribution uniformity ensures that green spaces are evenly distributed, making it easier for the older adult to access the natural environment, achieving a stress-relieving effect and reducing mental burden. Connectivity between green spaces provides continuous leisure and exercise routes, which can promote emotional regulation and reduce loneliness. Accessibility ensures that the older adult, especially those with limited mobility, can easily reach green spaces, promote their regular contact with nature, and help alleviate depression and anxiety.

## 3 Materials and methods

### 3.1 Data selection and preprocessing

CHARLS data collection adopts a multi-stage stratified sampling design, covering multiple provincial administrative regions in China, including urban and rural communities, to ensure the diversity of geographical, economic and demographic structures. To ensure the representativeness of the sample, the CHARLS team calibrated the sample weight based on the data of the sixth national census. This study further screened out 40 smart older adult care community samples. Data reliability is guaranteed by the following measures: the original questionnaire is collected by trained investigators at home, and the computer-assisted face-to-face interview system is used for real-time quality inspection; key variables are cross-validated through medical records and medical insurance data; missing values are filled by multiple imputation methods, and the imputation model includes 12 covariates such as age and gender to reduce bias.

To ensure the regional representativeness of the sample, this study conducted a stratified analysis of the regional composition of the sample based on the geographical distribution characteristics covered by the CHARLS data. The original sample covers 15 provincial administrative regions in the three major economic belts of eastern, central and western China, of which the eastern region accounts for 42.3% (1,015 people), the central region accounts for 35.7% (857 people), and the western region accounts for 22.0% (528 people).

This study strictly abides by the Declaration of Helsinki and the ethical standards of scientific research. To protect the privacy of the subjects, the original CHARLS data has been anonymized: personal identity information has been removed, random coding has been used to replace real community and individual identifiers, and sensitive variables have been blurred. The research team signed a data confidentiality agreement to ensure that only desensitized data is used during the analysis process, and the results do not involve any information that can be traced back to individuals.

The data of this study comes from CHARLS (46, 47), covering the health and behavioral characteristics of 2,400 older adult people from 2012 to 2020. It includes 40 retirement communities, involving dynamic surveys on the health and economic status of the older adult, providing high-quality longitudinal data support for this study.

Samples with a large number of missing data and invalid records, such as samples that did not answer the key health indicator questionnaire, were eliminated. According to the description of the types of residential communities where the older adult live in the sample, the sample group living in smart older adult care communities was screened out to ensure the high relevance of the research object and the research topic. Through the data screening step, the core sample data set suitable for this study was obtained.

The basic information of the older adult obtained is shown in Table 1.

**Table 1:** Basic information of the older adult.

**Feature**	**Category**	**Number of people**	**Percentage**
Gender	Male	1,200	50.0
	Female	1,200	50.0
Age	60–69	900	37.5
	70–79	1,100	45.8
	80 and above	400	16.7
Education level	Primary school and below	800	33.3
	Junior high school	1,000	41.7
	Senior high school and above	600	25.0
Marital status	Married	1,800	75.0
	Unmarried/divorced/widowed	600	25.0
Smoking status	Smoker	480	20.0
	Non-smoker	1,920	80.0
Drinking habits	Drinker	600	25.0
	Non-drinker	1,800	75.0
Frequency of physical exercise	Daily	540	22.5
	1–3 times a week	960	40.0
	Rarely	900	37.5

For each missing data, a Bayesian approach can be used to predict and interpolate missing values. m imputed data sets can be generated, and the missing values of each data set are interpolated as different estimates of possible values. Standard regression analysis is performed on each imputed data set to generate parameter estimates and standard errors for each data set.

The final estimates and standard errors are obtained by summarizing the estimation results of all imputed data sets. Combined estimates:


(1) $$\begin{array}{c} {\overset{ˆ}{\theta }}_{pool}=\frac{1}{m}\overset{m}{\underset{i=\;1}{\sum }}{\overset{ˆ}{\theta }}_{i} \end{array}$$

In formula 1, ${\overset{ˆ}{\theta }}_{pool}$ is the final combined estimate, and ${\overset{ˆ}{\theta }}_{i}$ is the estimate in the i-th interpolation dataset.

Combined standard error:


(2) $$\begin{array}{c} SE\left({\overset{ˆ}{\theta }}_{pool}\right)=\sqrt{\frac{1}{m}\overset{m}{\underset{i=1}{\sum }}\left(SE{\left({\overset{ˆ}{\theta }}_{i}\right)}^{2}+\frac{1}{m}{\left({\overset{ˆ}{\theta }}_{i}-\;{\overset{ˆ}{\theta }}_{pool}\right)}^{2}\right)} \end{array}$$

The combined t-statistic is:


(3) $$\begin{array}{c} t_{pool}=\;\frac{{\overset{ˆ}{\theta }}_{pool}}{SE\left({\overset{ˆ}{\theta }}_{pool}\right)} \end{array}$$

**Figure 1:**
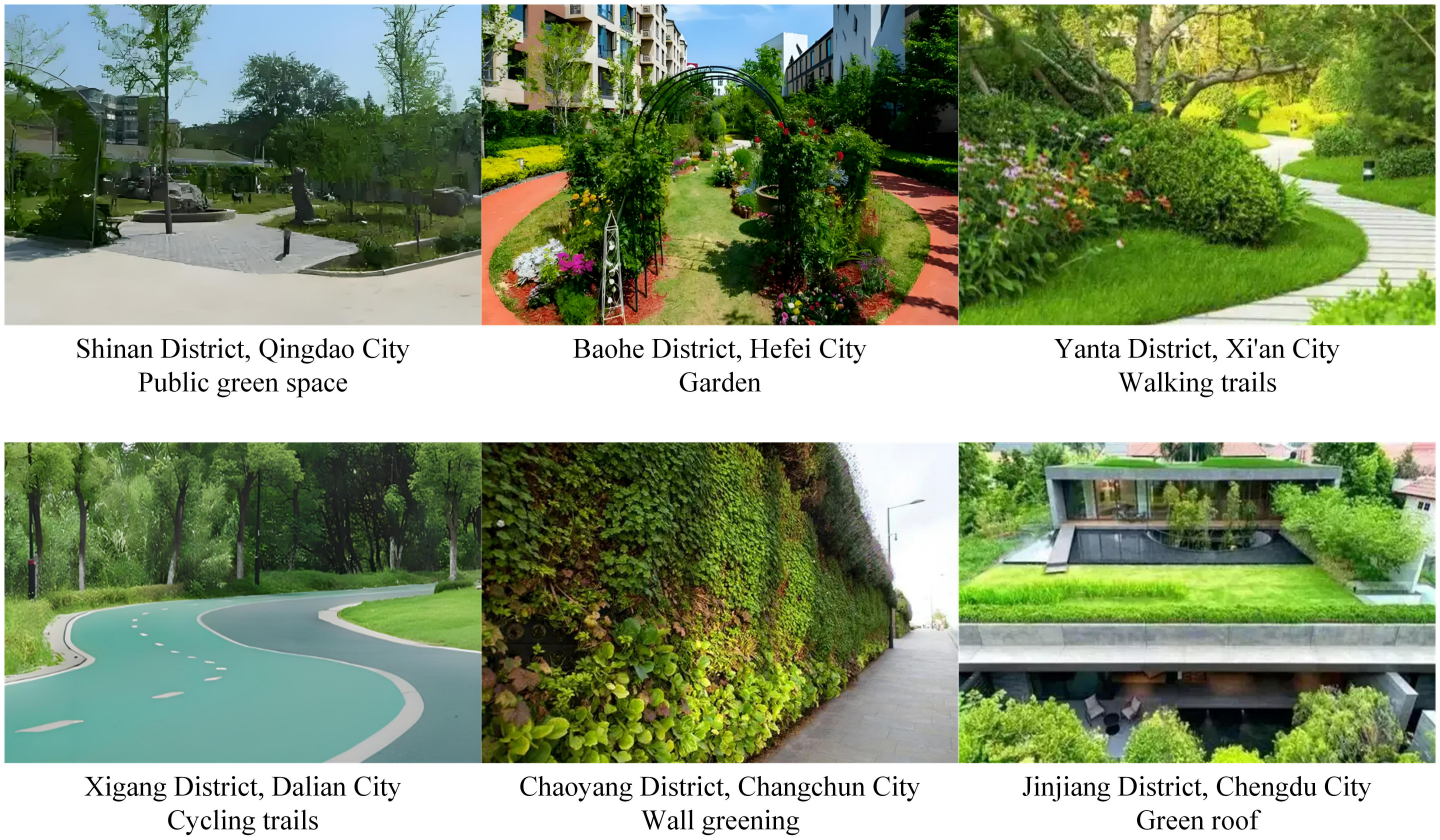
Green infrastructure. This figure shows six photographs of green infrastructure in retirement communities.

The green infrastructure of the retirement community is shown in Figure 1.

Green infrastructure in retirement communities, including public green spaces, gardens, walking paths, cycling paths, green walls and green roofs, has multiple positive effects. Public green spaces and gardens provide places for older adult people to relax, socialize and engage in physical activities. Walking paths and cycling paths encourage older adult people to engage in daily exercise. Wall greening and green roofs effectively improve air quality, regulate temperature, and enhance the comfort of the living environment. Green facilities optimize the environmental landscape, improve the beauty of the community, and provide a safer, more livable, and interactive living space for the older adult.

### 3.2 Determination and selection of indicators

In order to effectively measure the impact of green infrastructure in smart older adult care communities on the health of the older adult, three categories of indicators are selected, including green infrastructure indicators, older adult health indicators, and control variables.

Green infrastructure indicators include coverage, distribution uniformity, connectivity index, and accessibility. The coverage calculation formula is:


(4) $$\begin{array}{c} C=\frac{\sum NDVI_{i}\times A_{i}}{S} \end{array}$$

In Formula 4, *NDVI*_*i*_ is the normalized vegetation index of the i-th green space. *A*_*i*_is the area of the i-th green space.

The calculation formula of the Gini coefficient (48, 49) is as follows:


(5) $$\begin{array}{c} Gini=\frac{1}{2n^{2}\mu }\overset{n}{\underset{i=1}{\sum }}\overset{n}{\underset{j=1}{\sum }}|x_{i}-x_{j}| \end{array}$$

In Formula 5, *x*_*i*_ and *x*_*j*_ represent the green areas of the i-th and j-th regions, respectively, n is the total number of regions, and μ is the average green area of all regions. This indicator can be used to assess whether the spatial distribution of community green space resources is balanced, which in turn affects the convenience of use and social interaction opportunities for the older adult.

The connectivity index is used to measure the degree of connectivity of the green space network within the community, that is, whether the green space forms an interconnected space through trails, greenways, etc. The connectivity index formula is expressed as:


(6) $$\begin{array}{c} C_{new}=\frac{1}{\sum \left(L_{i}\cdot e^{\alpha \left(S_{i}+D_{i}+\;1/R_{i}\right)}\right)} \end{array}$$

Formula 6 makes weighted corrections to the walking obstacle coefficient (α) of the older adult, the comprehensive path slope (S), the obstacle density (D) and the rest node spacing (R).

older adult health indicators are divided into three categories: PH, MH, and social effects. The PH index is based on the data from the CHARLS questionnaire, which counts the types of chronic and acute diseases suffered by the older adult to assess the overall health of the individual. Chronic diseases include diabetes, hypertension, cardiovascular disease, etc., while acute diseases include infectious diseases or diseases that occur in a short period of time.

Each disease is scored as 1 point, and the cumulative value of the total score of all diseases reflects the health status of the individual. The formula is as follows:


(7) $$\begin{array}{c} DS=\overset{n}{\underset{i=\;1}{\sum }}D_{i} \end{array}$$

In Formula 7, *D*_*i*_ represents the score of suffering from the i-th disease (1 is suffering from the disease and 0 is not suffering from the disease).

MH is measured by the SDS scale, SAS scale, and SWB scale, which are used to assess the depression level, anxiety level, and subjective wellbeing of the older adult, respectively.

The SDS scale consists of 20 questions, and each question has a score range of 1–4 points, corresponding to “none or rarely”, “sometimes”, “often”, and “always”, respectively. The core of the scale to assess depressive symptoms includes mood, interest, sleep, appetite and physiological function. The total score ranges from 20 to 80 points. The evaluation criteria of the SDS scale are shown in Table 2.

**Table 2:** SDS scale.

**Scale**	**Score range**	**Depression status**
Self-Rating Depression Scale	20–39	No depressive symptoms
	40–47	Mild depression
	48–55	Moderate depression
	>56	Severe depression

The SAS scale consists of 20 questions, each with a score range of 1 to 4, representing “none or rarely”, “sometimes”, “often” and “always”. The scale focuses on the core symptoms of anxiety disorders, such as tension, fear, fatigue, insomnia, etc. The total score ranges from 20 to 80 points. The evaluation criteria of the SAS scale are shown in Table 3.

**Table 3:** SAS scale.

**Scale**	**Score range**	**Anxiety status**
Self-Rating Anxiety Scale	20–39	No anxiety symptoms
	40–47	Mild anxiety
	48–55	Moderate anxiety
	>56	Severe anxiety

The evaluation criteria of the SWB scale are shown in Table 4.

**Table 4:** SWB scale.

**Scale**	**Score range**	**Subjective wellbeing**
Subjective wellbeing	< 70	Low level
	70–104	Medium level
	>104	High level

The social impact index assesses the frequency of interaction between the older adult and their family, friends, and community members in their daily lives as a quantitative standard for their social support and social participation. The interaction frequency is measured in times/week. The interaction frequency is the sum of the number of times the older adult interact with different objects per week, and includes the following three parts:


(8) $$\begin{array}{c} f=F_{1}+F_{2}+F_{3} \end{array}$$

*F*_1_, *F*_2_, and *F*_3_ represent the number of weekly interactions between the older adult and their family members, the number of weekly interactions between the older adult and their friends, and the number of weekly interactions between the older adult and community members, respectively.

### 3.3 Regression analysis model

The panel data regression model can effectively control the unobservable heterogeneity at the individual level and consider the differences between time and individuals. The fixed effect model is used to control the unobservable heterogeneity factors of individuals, which remain unchanged at different time points. Through fixed effects, the influence of these time-invariant individual characteristics on the dependent variable can be removed, making the estimation results more accurate. The formula of the fixed effect model (50, 51) is:


(9) $$\begin{array}{c} Y_{it}=\beta X_{it}+\alpha_{i}+\;\epsilon_{it} \end{array}$$

In Formula 9, *Y*_*it*_ is the dependent variable, which is the health or social effect indicator of the i-th older adult person in period t. *X*_*it*_ is the coverage rate of green infrastructure.

REM (Random Effects Model) (52, 53) assumes that the unobservable heterogeneity of individuals is random and unrelated to the explanatory variables. The random effects model is more flexible in analysis and is suitable for data with large cross-temporal and cross-individual differences. The formula is:


(10) $$\begin{array}{c} Y_{it}=\beta X_{it}+u_{i}+\;\epsilon_{it} \end{array}$$

In Formula 10, *u*_*i*_ is the individual random effect, which is the unobservable difference between individuals. Unlike the fixed effect model, the random effect assumes that individual differences are independent of the explanatory variables, so that both time effect and individual effect can be considered in the model.

The basic form of the Hausman test is:


(11) $$\begin{array}{c} H=\left(b_{FE}-b_{RE}\right)\prime {\left[Var\left(b_{FE}\right)-Var\left(b_{RE}\right)\right]}^{-1}\left(b_{FE}-b_{RE}\right) \end{array}$$

In order to verify the robustness of the model results, the following two methods are used for testing:

Shorten the time window: The time window can be shortened to examine the impact of changes in time periods on the results. This helps to eliminate trend bias caused by long time span.
Eliminate some samples: Special groups (older people over 80 years old) can be excluded for sensitivity analysis to ensure the representativeness of the sample and check whether there is a SI (significant impact) of specific groups on the model results.

To further explore the differences in the impact of green infrastructure on health and social effects among different groups, heterogeneity tests are conducted. The analysis was performed by gender, age, and education level. Regression analysis of different subgroups was performed to compare the differences in the impact of green infrastructure in each group, thereby revealing possible heterogeneous effects.

### 3.4 Health impacts on the older adult

The cross-sectional study collects data from older adult groups in different communities from June 2020 to June 2021 to analyze the differences in the prevalence of COVID-19 among the older adult in communities with different levels of green infrastructure coverage. Data collection will be based on factors such as the health status of the older adult in the community, the COVID-19 infection rate, and green infrastructure data. Health data and green space coverage data of the older adult are collected through questionnaires, community health records, and COVID-19 epidemic reports provided by the government.

SEM will be used to analyze the impact of green infrastructure on the prevalence of COVID-19 among the older adult and verify the interrelationships between various green infrastructure variables. The SEM model can estimate multiple causal relationships at the same time and analyze the direct effects between variables. The following is the basic construction of the SEM model:

Independent variables include green space coverage, green space distribution uniformity, green space connectivity, green space accessibility.
Dependent variable: COVID-19 prevalence.

In SEM, a measurement model is used to describe the relationship between observed indicators and latent variables. Its basic form is:


(12) $$\begin{array}{ccc} y & = & \Delta_{y}\eta +\;\epsilon \end{array}$$

(13) $$\begin{array}{ccc} x & = & \Delta_{x}\xi +\;\delta \end{array}$$

In Formulas 12, 13, y is the COVID-19 prevalence, and x is the independent variable, including green space coverage, distribution uniformity, connectivity, and accessibility. η and ξ are the latent variables of the dependent variable and independent variable, respectively.

The structural model is used to describe the causal relationship between latent variables, and its basic expression is:


(14) $$\begin{array}{c} \eta =B\eta +\Gamma \xi +\;\zeta \end{array}$$

In Formula 14, B is the interaction matrix between the dependent variable and the latent variable, Γ is the direct effect matrix of the independent variable on the dependent variable, and ζ is the structural error term.

In this paper, the mediating effect analysis of green infrastructure optimization on the prevalence of COVID-19 is carried out, and the indicators of green infrastructure are used as independent variables, and air quality and ambient temperature are used as mediating variables to construct the following mediation model:


(15) $$\begin{array}{c} M_{1}=a_{1}X+\;\varepsilon_{M_{1}} \end{array}$$

(16) $$\begin{array}{c} M_{2}=a_{2}X+\;\varepsilon_{M_{2}} \end{array}$$

In Formulas 15, 16, *M*_1_ and *M*_2_ represent air quality and ambient temperature, respectively, and *a*_1_ and *a*_2_ are the impact coefficients of green infrastructure on mediating variables.

The resulting model is:


(17) $$\begin{array}{c} Y=c\prime X+b_{1}M_{1}+b_{2}M_{2}+\;\varepsilon_{Y} \end{array}$$

In Formula 17, Y is the COVID-19 prevalence rate, and *c*′ is the direct effect of green infrastructure on the COVID-19 prevalence rate.

## 4 Empirical analysis results

### 4.1 Impact of green infrastructure coverage on chronic and acute diseases among the older adult

**Figure 2:**
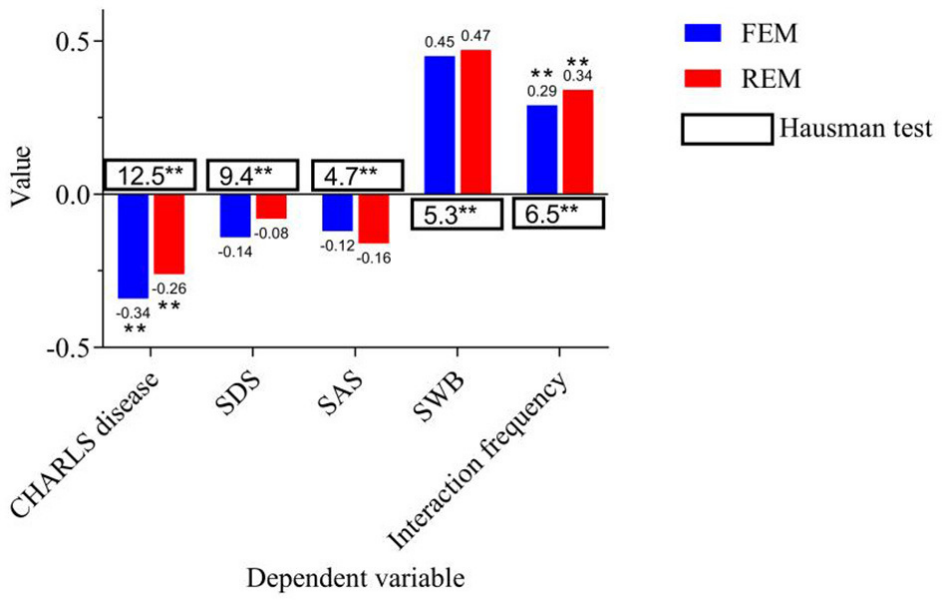
Panel data regression analysis. *, **, *** indicate *p* < 0.05, *p* < 0.01, *p* < 0.001, respectively.

Fixed effects and random effects were used for fitting, and the regression analysis results are shown in Figure 2.

In Figure 2, the independent variable is coverage. For the dependent variables “CHARLS disease” and “interaction frequency”, green infrastructure coverage shows a SI in both Fixed Effects Model (FEM) and Random Effects Model (REM). The fixed effect coefficient of CHARLS disease is −0.34 and significant (*p* < 0.01), while the random effect coefficient is −0.26, which is also significant (p < 0.01). This shows that smart older adult care communities with a high coverage of green infrastructure can help significantly reduce the incidence of chronic and acute diseases among the older adult. For the dependent variable “interaction frequency”, the coefficients of the fixed effect and random effect are 0.29 and 0.34 respectively, both of which are significantly positively correlated (p < 0.01). This further supports that the improvement of green infrastructure helps promote interaction between the older adult and others, and enhances their sense of social participation and social support.

In this study, the fixed effect model can better control the heterogeneity at the individual level and remove the bias caused by individual differences, so it can provide more robust estimation results. For CHARLS diseases, both the fixed effect and random effect models show that the coverage rate of green infrastructure has a negative impact on the incidence of chronic and acute diseases among the older adult. It indicates that smart older adult care communities with higher coverage rates can effectively reduce the occurrence of health problems among the older adult, and hypothesis 1 has been verified. The coverage of green infrastructure is significantly positively correlated with the frequency of daily interactions among the older adult (fixed effect coefficient is 0.29, random effect coefficient is 0.34), and the results of the Hausman test further support the fixed effect model.

### 4.2 The impact of green infrastructure improvement on the social interaction frequency of the older adult

**Figure 3:**
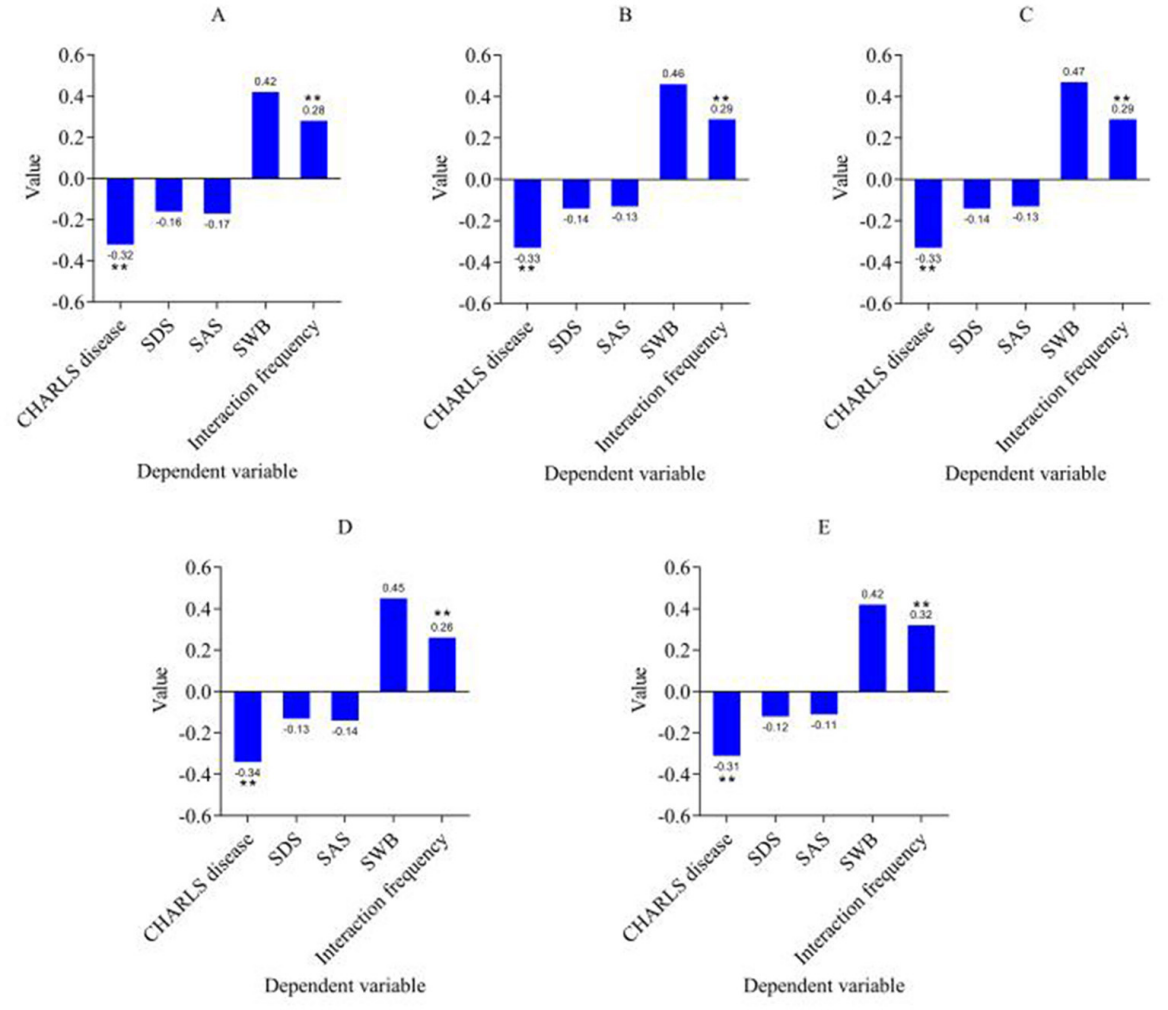
Robustness test results. **(A)** Time window is 2012–2014. **(B)** Time window is 2014–2016. **(C)** Time window is 2016–2018. **(D)** Time window is 2018–2020. **(E)** Eliminating people over 80 years old. ^***^p < 0.001, ^**^p < 0.01, ^*^p < 0.05.

By shortening the time window, it can check whether the model results are consistent in different time periods. Eliminating extremely special groups (people over 80 years old) can help eliminate possible extreme effects and avoid interference of special groups on the overall analysis results. Through robustness testing, the paper confirms whether the model results are widely applicable and reliable. The time window is shortened to 2012–2014, 2014–2016, 2016–2018, and 2018–2020. The robustness test results are shown in Figure 3.

According to the robustness test results (shortening the time window and excluding samples of people over 80 years old), it is observed that the impact of green infrastructure coverage on the PH and social effects of the older adult is still significantly stable. The regression analysis results show that the shortening of the time window (2012–2014, 2014–2016, 2016–2018, 2018–2020) and the exclusion of samples over 80 years old. The impact of green infrastructure on chronic diseases (CHARLS diseases) and social effects (interaction frequency) remains stable and significant. The coefficient of CHARLS diseases is always negative and significant.

The robustness test results further verified the role of green infrastructure in promoting the social interaction frequency of the older adult. Although the coefficients of SDS, SAS, and SWB fluctuated in different time windows, they still showed that the coverage of green infrastructure had a relatively small impact on the MH and subjective wellbeing of the older adult (the coefficients of SDS and SAS fluctuated slightly, and neither reached a significant level). The frequency of interaction continued to show a positive correlation, and the results were basically consistent in different time windows and after excluding special groups. In particular, the coefficient of interaction frequency is always positive and significant in all regression models. This shows that the improvement of green infrastructure can effectively promote the interaction frequency between the older adult and others, further verifying the validity of hypothesis 2.

In summary, the robustness test results still confirm the improvement effect of green infrastructure on the health status of the older adult after shortening the time window and eliminating special groups. In policy and practice, expanding the coverage of green infrastructure construction can become a key way to improve the wellbeing of the older adult.

### 4.3 The impact of optimized green infrastructure design on the mental health of the older adult

**Figure 4:**
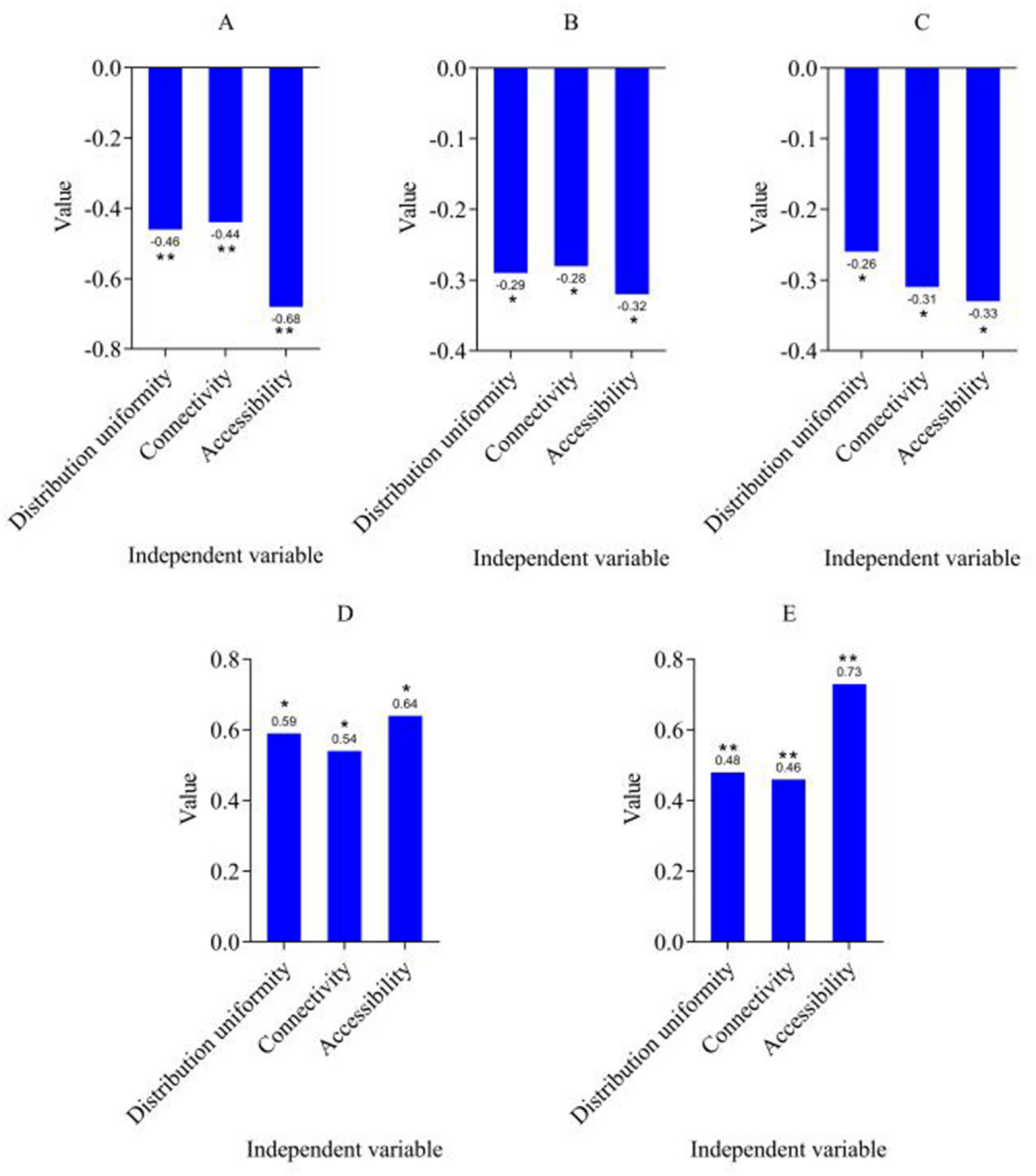
Pathway optimization results. **(A)** CHARLS disease. **(B)** SDS. **(C)** SAS. **(D)** SWB. **(E)** Interaction frequency. ^***^p < 0.001, ^**^p < 0.01, ^*^p < 0.05.

By introducing green space distribution uniformity, connectivity and accessibility as key independent variables, the green infrastructure path of the older adult community is optimized to explore its specific impact on the health and social effects of the older adult. The results are shown in Figure 4.

Green space distribution uniformity, connectivity and accessibility are used as independent variables by introducing three key indicators. The regression results of the CHARLS disease indicators showed that green space accessibility had the most SI on the incidence of chronic diseases among the older adult (the regression coefficient was −0.68), and the uniformity of green space distribution and connectivity also had a SI on the improvement of health status (−0.46 and −0.44, respectively). Regarding the measurement of MH, the regression coefficients of the two indicators SDS and SAS are both negative, and are significant under the three independent variables (SDS is −0.29, −0.28, −0.32, SAS is −0.26, −0.31, −0.33). This shows that with the optimization design of green infrastructure, the depression and anxiety of the older adult have been effectively alleviated, especially the accessibility of green space has the most significant effect on improving MH. Hypothesis 3 was verified, that is, the optimized design of green infrastructure can effectively alleviate depression and anxiety among the older adult.

In terms of subjective wellbeing, the regression coefficients of SWB are also positive and are significant under the three independent variables (green space distribution uniformity is 0.59, connectivity is 0.54, and accessibility is 0.64). This shows that the optimized green infrastructure significantly improves the subjective wellbeing of the older adult, especially the accessibility of green space has the most prominent effect on improving wellbeing. With the optimization of green infrastructure, the quality of life and social participation of the older adult have been enhanced, further improving their subjective wellbeing. As a social effect indicator, the regression coefficients of interaction frequency under the three independent variables are also positive, and it shows the greatest impact on green space accessibility (the regression coefficient is 0.73). This result shows that the optimized green infrastructure improves the physical and MH of the older adult and promotes the interaction frequency between the older adult and others, enhances the level of social support and social participation, and thus improves the overall wellbeing of the older adult.

### 4.4 Heterogeneity analysis of hypothesis verification results

The heterogeneity results based on gender are shown in Table 5.

**Table 5:** Heterogeneity results based on gender.

**Dependent variable**	**Independent variable: coverage**	
	**Gender**	
	**Male**	**Female**
CHARLS disease	−0.42^**^	−0.31^**^
Self-Rating Depression Scale	−0.17	−0.11
Self-Rating Anxiety Scale	−0.19	−0.07
Subjective wellbeing	0.52	0.44
Interaction frequency	0.32^**^	0.26^**^

^***^p < 0.001, ^**^p < 0.01, ^*^p < 0.05.

There are some differences in the impact of green infrastructure coverage on the health and social effects of male and female older adult people. In the MH indicators (SDS and SAS), the regression coefficients of men are larger than those of women (−0.17 for men and −0.11 for women. The regression coefficient of green infrastructure on the interaction frequency of male older adult people is −0.19 for males and −0.07 for females), which may indicate that green infrastructure has a more significant effect on improving the MH of male older adult people, especially in terms of anxiety and depression. The regression coefficient of SWB showed a positive correlation for both sexes, with the regression coefficient of males (0.52) slightly higher than that of females (0.44). This shows that the improvement of green infrastructure has a more significant effect on the subjective wellbeing of men, which may be related to the stronger environmental dependence and physical and mental needs of male older adult people. Although green infrastructure has a positive impact on both male and female older adult people, men have better responses in terms of chronic disease prevention, MH improvement, and social participation. This provides important empirical evidence for considering gender differences when formulating green infrastructure policies for older adult communities.

The results of heterogeneity based on age are shown in Table 6.

**Table 6:** Heterogeneity based on age.

**Dependent variable**	**Independent variable: Coverage**		
	**Age**		
	**60–69**	**70–79**	**80 and above**
CHARLS disease	−0.35^**^	−0.38^**^	−0.34^**^
Self–Rating Depression Scale	−0.15	−0.19	−0.14
Self–Rating Anxiety Scale	−0.12	−0.18	−0.12
Subjective wellbeing	0.43	0.49	0.45
Interaction frequency	0.27^**^	0.35^**^	0.29^**^

^***^p < 0.001, ^**^p < 0.01, ^*^p < 0.05.

Judging from the CHARLS disease indicators, the older adult aged 60–69, 70–79, and 80 years and above all showed negative relationships, and all reached a significant level, indicating that the improvement of green infrastructure has a significant effect on the prevention of chronic diseases in the older adult of all ages. For the MH index, the regression coefficients of SDS and SAS are both negative, indicating that green infrastructure can alleviate depression and anxiety, but the difference between different age groups is not significant. For the 70–79 age group, the regression coefficients of SDS and SAS are −0.19 and −0.18, respectively, both of which are negative, indicating that the MH of the older adult in this age group is greatly affected by the improvement of green infrastructure. For the social effect index, the regression coefficients of all age groups are positive and statistically significant, indicating that green infrastructure can significantly increase the social interaction frequency of the older adult. In terms of interaction frequency, the regression coefficient (0.35) of the 70–79 age group is the highest. This means that the increase in social interaction frequency promoted by the improvement of green infrastructure for the older adult in this age group is the most obvious, which may be related to the higher demand of the older adult in this age group for a social environment.

The heterogeneity results based on education level are shown in Table 7.

**Table 7:** Heterogeneity based on education level.

**Dependent variable**	**Independent variable: coverage**		
	**Education level**		
	**Primary school and below**	**Junior high school**	**Senior high school and above**
CHARLS disease	−0.33^**^	−0.34^**^	−0.34^**^
Self–Rating Depression Scale	−0.15	−0.14	−0.14
Self–Rating Anxiety Scale	−0.12	−0.12	−0.12
Subjective wellbeing	0.45	0.45	0.45
Interaction frequency	0.28^**^	0.29^**^	0.29^**^

^***^p < 0.001, ^**^p < 0.01, ^*^p < 0.05.

The CHARLS disease index showed a significant negative relationship in all education level groups, with regression coefficients of −0.33, −0.34 and −0.34, respectively, and statistically significant, indicating that the improvement of green infrastructure can effectively reduce the incidence of chronic diseases in the older adult, regardless of their educational background. The regression coefficients of SDS and SAS in different education level groups are slightly different, both of which are negative, indicating that green infrastructure improvement has a positive impact on alleviating the MH problems of the older adult, and the impact is relatively consistent among different education level groups. Green infrastructure has a relatively stable effect on improving MH in all groups, and its regression coefficients are close in all groups, indicating that education level has no significant moderating effect on this impact. The regression coefficients of SWB and interaction frequency also showed similar trends in each education group, all of which were positive. In summary, regardless of the education level of the older adult, green infrastructure has a relatively consistent effect on their health status, MH, and social interaction frequency, indicating that the heterogeneity of education level in such studies is small, and the improvement of green infrastructure has a positive impact on the older adult of all educational backgrounds.

The heterogeneous effects of gender, age and education level found in this study may be closely related to differences in social and cultural norms and intergenerational resource allocation. In terms of gender, male older adult people benefit more from green infrastructure (CHARLS disease coefficient −0.42^**^ vs. female −0.31^**^), which may be due to the fact that males use public spaces more frequently (such as morning exercises and social activities) under traditional gender roles, while females use them less frequently due to family care responsibilities or safety concerns. In the age stratification, the social frequency of the 70–79 age group increased most significantly (coefficient 0.35^**^), reflecting that this group is in the “active aging” stage, with both high social needs and good physical function to participate in outdoor activities, while the older adult (80+) have limited mobility, and the accessibility of green space has a weakened effect on their health promotion. The heterogeneity of education level is not significant (the difference in coefficients among groups < 0.01), which may be due to the fact that the inclusive policy of China's smart older adult care community has weakened the barrier of educational resources to the acquisition of health resources, but it should be noted that the low-education group may not fully transform the health benefits of green space due to insufficient health literacy.

### 4.5 The impact of community green spaces on COVID-19 in the older adult

A cross-sectional study was conducted to calculate the prevalence of COVID-19 among the older adult in the community. The data are from 2019 to 2020 and the results are shown in Table 8.

**Table 8:** Prevalence of COVID-19 among the older adult in the community.

**Years**	**Green space coverage (%)**	**COVID-19 prevalence (%)**
2019	< 10	15.2
	10–20	12.8
	20–30	10.6
	30–40	8.3
	>40	5.1
2020	< 10	18.7
	10–20	14.5
	20–30	11.4
	30–40	9.1
	>40	6.2

Through a cross-sectional study, the relationship between the prevalence of COVID-19 among the older adult in the community and the green space coverage rate from 2019 to 2020 was analyzed, and it was found that the green space coverage rate and the prevalence of COVID-19 showed a significant negative correlation trend. Specifically, in 2020, in communities with a green space coverage rate of more than 40%, the prevalence of COVID-19 among the older adult was 6.2%, which was much lower than that in communities with a green space coverage rate of < 10% (18.7%). This trend was also reflected in 2019. The prevalence of COVID-19 in communities with higher green space coverage (>40%) was 5.1%, which was significantly lower than the 15.2% in communities with lower green space coverage (< 10%). As the green space coverage rate went from low to high, the prevalence of COVID-19 in the older adult population in the community gradually decreased, showing the potential role of green space in reducing the risk of virus transmission and improving the health of the older adult.

This trend may be related to many factors. Communities with high green space coverage can provide better air quality, larger open spaces, and better social interaction environments, which help to enhance the immunity and physical health of the older adult population, thereby reducing the risk of disease infection. Communities with rich green spaces can usually promote moderate outdoor activities such as walking and morning jogging for the older adult, enhance the body's resistance to disease, and reduce psychological stress and immune system decline caused by long-term living in a closed environment. The increase in green space may lead to more social interactions among the older adult, help reduce loneliness and social isolation, which is also an important factor in improving the overall health of the older adult. On the contrary, in communities with low green space coverage, especially in the early stages of the COVID-19 outbreak, the older adult population often faces more closed living spaces and poor air quality, and due to the lack of outdoor activity venues suitable for the older adult, the older adult population has fewer opportunities for social activities and physical exercise, which may increase the risk of infection. By comparing the data from 2019 and 2020, the overall COVID-19 prevalence in 2020 has increased, which may be related to the spread of the epidemic and changes in prevention and control measures, but the green space coverage rate still shows its important role in epidemic prevention and control, especially in alleviating health risks for the older adult. In summary, the COVID-19 prevalence rate among the older adult in communities with high green space coverage is significantly lower, indicating that green infrastructure plays a positive role in improving the health protection and quality of life of the older adult. This provides strong support for strengthening green infrastructure construction in urban and community planning, especially in terms of health protection for the older adult. Increasing green space coverage can be used as an important public health intervention measure.

Green infrastructure can significantly reduce the load of respiratory pathogens through vegetation adsorption of PM_2.5_, filtering NOx and releasing negative oxygen ions. Green space can alleviate the urban heat island effect through transpiration and shading effect, while the suitable temperature (18–25°C) can inhibit viral activity and reduce the decline in immunity caused by heat stress in the population.

By introducing mediating variables such as air quality and ambient temperature, SEM was used to analyze the impact of coverage, green space distribution uniformity, green space connectivity, and green space accessibility on the prevalence of COVID-19. The path coefficient estimation results are shown in Table 9.

**Table 9:** Path coefficient estimation results.

**Path**	**Unstandardized value**	**Standardized value**	**Standard error**	**Z value**	***P* value**
Green space coverage → Air quality → COVID-19 prevalence	−0.2	−0.15	0.05	−4	< 0.001
Green space coverage → Ambient temperature → COVID-19 prevalence	−0.12	−0.1	0.04	−3	< 0.01
Green space distribution uniformity → Air quality → COVID-19 prevalence	−0.18	−0.13	0.05	−3.6	< 0.001
Green space distribution uniformity → Ambient temperature → COVID-19 prevalence	−0.11	−0.09	0.04	−2.8	< 0.01
Green space connectivity → Air quality → COVID-19 prevalence	−0.16	−0.12	0.05	−3.2	< 0.01
Green space connectivity → Ambient temperature → COVID-19 prevalence	−0.1	−0.08	0.04	−2.5	< 0.05
Green space accessibility → Air quality → COVID-19 prevalence	−0.25	−0.18	0.06	−4.5	< 0.001
Green space accessibility → Ambient temperature → COVID-19 prevalence	−0.15	−0.11	0.05	−3.5	< 0.001
Direct effect: Independent variable → COVID-19 prevalence	−8	−0.2	0.07	−3.8	< 0.001

Green space coverage, green space distribution uniformity, green space connectivity and green space accessibility can significantly improve air quality and regulate ambient temperature, thereby indirectly reducing the risk of COVID-19 infection in the older adult. The improvement of air quality and ambient temperature by green space coverage was transmitted to the COVID-19 prevalence, and the standardized path coefficients were −0.15 and −0.10, respectively, both reaching statistical significance (P < 0.01), indicating that increasing the proportion of community green space helps to reduce the risk of the epidemic. The standardized effects of green space distribution uniformity in the air quality and ambient temperature paths were −0.13 and −0.09, respectively, indicating that balanced resource distribution can further optimize environmental conditions. Green space connectivity and accessibility showed relatively significant negative effects through two mediating paths, among which the standardized effect of green space accessibility transmitted through air quality was −0.18, which further emphasized the importance of convenient green space access to improve the community microenvironment. In addition to the indirect effect, the independent variable still has a significant direct negative effect on the prevalence of COVID−19 (standardized value = −0.20, P < 0.001), indicating that the existence of the green space system itself also plays a positive role in reducing the risk of infection. Overall, these results verify the mediating effect of green infrastructure in reducing the risk of COVID-19 by improving air quality and regulating ambient temperature and emphasize the importance of optimizing green space layout to improve public health and safety, and provide solid empirical support for the planning and construction of smart older adult care communities and epidemic prevention and control policies.

The results of factor path and effect analysis are shown in Table 10.

**Table 10:** Results of factor path and effect analysis.

**Path**	**Direct effect**	**Indirect effect**	**Total effect**	**Indirect effect ratio**	**Bootstrap 95% CI**
Green cover → Air quality → COVID-19	−0.08	−0.15	−0.23	65.20%	[−0.25,−0.08]
Green cover → Temperature regulation → COVID-19	−0.06	−0.1	−0.16	62.50%	[−0.18, −0.04]
Evenness of distribution → Air quality → COVID-19	−0.05	−0.13	−0.18	72.20%	[−0.30, −0.10]
Evenness of distribution → Temperature regulation → COVID-19	−0.03	−0.09	−0.12	75.00%	[−0.22, −0.06]
Connectivity → Air quality → COVID-19	−0.04	−0.12	−0.16	75.00%	[−0.28, −0.09]
Connectivity → Temperature regulation → COVID-19	−0.02	−0.08	−0.1	80.00%	[−0.20, −0.05]
Accessibility → Air quality → COVID-19	−0.07	−0.18	−0.25	72.00%	[−0.35, −0.15]
Accessibility → Temperature regulation → COVID-19	−0.04	−0.11	−0.15	73.30%	[−0.24, −0.10]

This study used SEM to construct a dual mediation path model of the impact of green infrastructure on the prevalence of COVID-19, and tested the robustness of each path through Bootstrap sampling. Whether it is green space coverage, green space distribution uniformity, green space connectivity or green space accessibility, these four indicators have significant indirect effects on the prevalence of COVID-19 by improving air quality and regulating ambient temperature. The standard errors of each path are small and the Bootstrap 95% confidence interval does not cross zero, which fully proves the statistical significance of the mediation effect. Among them, the effect of green space accessibility transmitted through the air quality and ambient temperature paths is particularly significant, with the lower and upper limits of the confidence interval reaching −0.35 and −0.15 respectively. It indicates that convenient green space access has a more prominent role in optimizing the community microenvironment and preventing and controlling the epidemic; while green space coverage and distribution uniformity also play a negative transmission effect on the two mediation paths, suggesting that increasing the proportion of green space and balanced layout can directly reduce the risk of COVID-19 infection and indirectly inhibit the spread of the virus by improving environmental conditions; green space connectivity plays a relatively stable mediating role in improving air quality. Overall, the results systematically reveal the key role of green infrastructure in reducing the prevalence of COVID-19 among the older adult through environmental regulation mediating effects, providing empirical evidence for the planning of smart older adult care communities and public health prevention and control, and emphasizing that in urban planning and community construction. Attention should be paid to the all-round optimization of green space systems, so as to effectively improve the health level and anti-epidemic ability of the older adult by improving air quality and microclimate environment.

## 5 Discussions

Green infrastructure with a high coverage rate significantly reduces the risk of illness among the older adult, especially in terms of health indicators such as CHARLS disease, and the health status is effectively improved. Green space can reduce air pollution, regulate temperature, and provide a quiet environment, thereby reducing the psychological pressure and physiological burden of the older adult and reducing the incidence of diseases. Hypothesis 2 enhances the social participation and social support level of the older adult, and this hypothesis was also verified. By increasing the coverage of community green space, the older adult can more conveniently participate in community activities, enhance interaction between neighbors, and improve their sense of social support and participation. This mechanism reflects how environmental factors affect the establishment of social relationships and the social-emotional state of the older adult. The verification results of hypothesis 3 further confirmed that the optimal design of green infrastructure, including distribution uniformity, connectivity and accessibility, can effectively alleviate depression and anxiety among the older adult and improve their subjective wellbeing.

The impact of urban green space on the health of the older adult has been verified worldwide. Urban green space helps improve the physical health of the older adult and effectively improves mental health and social interaction. older adult people with close social networks use urban parks more frequently, which in turn improves their mental health (54, 55). Similarly, other studies have reviewed relevant global literature and demonstrated a positive correlation between urban green space and subjective wellbeing in the older adult (56). These studies show that the positive impact of urban green space on the mental health of the older adult is common in different countries, but its effect is affected by cultural and social background.

In addition, studies have shown that the perception of green space by the older adult is significantly related to their health status, especially when green space accessibility is high, the physical and mental health of the older adult is effectively improved (57). However, other studies have found that the increase in green space may not significantly improve the mental health of the older adult, indicating that the effect of urban green space may need to be combined with other factors, such as social support and medical services (58).

Although existing studies have demonstrated the health benefits of urban green space, differences in different regions and cultures still affect the effectiveness of green space use (7). The cultural and socioeconomic backgrounds of different countries and regions may lead to different needs and uses of green space by the older adult. Therefore, the design of urban green spaces should be adapted to local conditions and fully consider the actual needs of the older adult population in order to achieve better health promotion effects (59, 60).

Green space coverage, distribution uniformity, connectivity index, and accessibility have significant effects on COVID-19 prevalence, indicating that improving community green infrastructure helps reduce the risk of disease transmission and promotes the physical and mental health of the older adult. Therefore, public health interventions should first focus on improving the accessibility and quality of green spaces, especially in communities where the older adult live densely. Increasing green space coverage can provide more outdoor activity space for the older adult and reduce the negative impacts of closed environments on health, such as air pollution and social isolation, thereby effectively reducing the risk of disease transmission and the incidence of chronic diseases. In addition, evenly distributed green space resources help ensure that every community member, especially the older adult, can equally enjoy the health benefits of green space and avoid health inequalities caused by the lack of green space in some areas. Connectivity is also an important aspect of optimizing green space design, ensuring that green spaces form an interconnected network through facilities such as trails and greenways, so that the older adult can easily get from one green space area to another, increase their outdoor activity opportunities and social interactions, and thus improve their mental health and physical immunity. Public health interventions should also include health education and policy support, such as encouraging the older adult to participate in outdoor sports, organizing health checks and epidemic prevention publicity activities, etc., to help the older adult group understand how to use green space resources to improve their health (61–63). In terms of epidemic prevention and control, optimizing green space design can help reduce the risk of virus transmission and improve the social participation and life satisfaction of the older adult, thereby improving their overall health level. Therefore, comprehensively considering the coverage, distribution uniformity, connectivity and accessibility of green space, combined with public health intervention strategies, can effectively promote the health of the older adult. Especially in the context of the global epidemic, optimizing community green space design provides a practical solution for public health management.

### 5.1 Policy implementation

#### 5.1.1 Short-term measures (1–3 years)

Optimize accessibility: Add barrier-free passages and rest nodes (such as seats and awnings) in existing communities to increase the utilization rate of green space for the older adult (need collaboration between municipal departments and communities, low cost and quick results).

Smart monitoring deployment: Use IoT sensors to monitor air quality, temperature and humidity in real time, and give priority to covering high-density older adult communities (relying on existing smart city infrastructure, pilot projects can be carried out within 6 months).

Activate temporary green space: Convert idle plots into micro green spaces (such as pocket parks), combined with community volunteer maintenance (refer to the “greening wherever there is space” model in Jing'an District, Shanghai, with an implementation period of 2-3 months).

#### 5.1.2 Long-term measures (5–10 years)

Reconstruct spatial planning: Incorporate green infrastructure into the overall urban plan, and require that the green space coverage rate of newly built older adult communities be ≥35% (need to revise the “Urban Residential Area Planning and Design Standards” and coordinate with natural resources and housing and construction departments).

Eco-medical collaborative network: Establish a linkage mechanism between green space and community health service centers (such as combining rehabilitation gardens with chronic disease management), which requires cross-domain collaboration between health departments and urban planning agencies.

Introduction of social capital: Attract enterprises to participate in the construction of green older adult care communities through the PPP model, and provide supporting tax and land policy incentives (refer to the “government + enterprise + NGO” cooperation paradigm in Tianhe District, Guangzhou).

This study recommends integrating the green infrastructure system into the urban aging-friendly planning framework, achieving the hard indicators of green space coverage ≥40% and 5-minute accessibility 100% through a three-dimensional space optimization model, and building an “ecological-health-service” composite older adult care community by combining IoT environmental monitoring and AI health early warning systems. Focus on promoting the application of roof greening and vertical forest technology (≥30% building facade greening rate), supporting aging-friendly trails (slope ≤ 5%), smart rest nodes (health monitoring seats are set up every 200 meters), and establishing a linkage mechanism between community green space and medical resources. It is recommended that the natural resources department add a “silver hair green view rate” indicator (≥35%) in the control detailed plan, encourage developers to participate through policies such as floor area ratio rewards, and form a replicable healthy aging city planning paradigm.

Through these comprehensive measures, a better living environment can be provided for the older adult, their physical and MH can be improved, their quality of life can be improved, and the sustainable development of smart older adult care communities can be promoted. Improving green space coverage and accessibility is highly feasible in many cities, especially through land use policies, urban planning and financial support. However, policy implementation needs to consider economic costs and urban space limitations, so it is necessary to give priority to high-density and aging areas for green space expansion. At the same time, health impact assessments should include long-term monitoring of health changes in the older adult population after green space transformation, especially health data and mental health levels related to COVID-19. Public health interventions should be combined with green space planning to promote community activities, health education and physical exercise for the older adult. However, this study has certain limitations. First, the limitations of cross-sectional data cannot reveal causal relationships; second, the research data mainly come from specific communities and may not be widely representative. These findings should be further verified through larger-scale longitudinal studies in the future.

## 6 Conclusions

The results show that a higher coverage rate of green infrastructure can effectively reduce the incidence of chronic and acute diseases among the older adult, improve their overall health, and enhance their social participation and social support. By introducing factors such as green space distribution uniformity, connectivity, and accessibility, this study further confirms that the optimal design of green infrastructure can effectively alleviate depression and anxiety among the older adult and improve their subjective wellbeing. This study systematically explores the impact of green infrastructure on the physical and MH of the older adult from the perspective of green infrastructure, and provides new theoretical basis and empirical support for the planning and design of smart older adult care communities.

This study also has certain limitations, mainly reflected in the regional restrictions of the data and the locality of the research sample, which fails to cover more older adult groups in different regions. Future research can further expand the sample range and combine more influencing factors for in-depth analysis. In addition, with the development of smart technology, future research can explore more intelligent green infrastructure optimization solutions and its deeper mechanisms in the physical and MH of the older adult, in order to provide more targeted theoretical and practical guidance for the further development of smart older adult care communities. Future research can combine the Internet of Things and AI technologies to develop a real-time monitoring system for green spaces, dynamically optimize layout and resource allocation, and improve the accuracy of health interventions in smart older adult care communities.

## Data Availability

The original contributions presented in the study are included in the article/supplementary material, further inquiries can be directed to the corresponding author.
